# Indole Propionic Acid Increases T Regulatory Cells and Decreases T Helper 17 Cells and Blood Pressure in Mice with Salt-Sensitive Hypertension

**DOI:** 10.3390/ijms24119192

**Published:** 2023-05-24

**Authors:** Gaurav Baranwal, Bethany L. Goodlett, Cristina M. Arenaz, Heidi A. Creed, Shobana Navaneethabalakrishnan, Joseph M. Rutkowski, Robert C. Alaniz, Brett M. Mitchell

**Affiliations:** 1 Department of Medical Physiology, Texas A&M University School of Medicine, Bryan, TX 77807, USAbethany.goodlett@tamu.edu (B.L.G.);; 2 Department of Microbial Pathogenesis and Immunology, Texas A&M University School of Medicine, Bryan, TX 77807, USA

**Keywords:** hypertension, kidney, microbiome, immunity, Treg, Th17

## Abstract

Hypertension affects over a billion adults worldwide and is a major risk factor for cardiovascular disease. Studies have reported that the microbiota and its metabolites regulate hypertension pathophysiology. Recently, tryptophan metabolites have been identified to contribute to and inhibit the progression of metabolic disorders and cardiovascular diseases, including hypertension. Indole propionic acid (IPA) is a tryptophan metabolite with reported protective effects in neurodegenerative and cardiovascular diseases; however, its involvement in renal immunomodulation and sodium handling in hypertension is unknown. In the current study, targeted metabolomic analysis revealed decreased serum and fecal IPA levels in mice with L-arginine methyl ester hydrochloride (L-NAME)/high salt diet-induced hypertension (LSHTN) compared to normotensive control mice. Additionally, kidneys from LSHTN mice had increased T helper 17 (Th17) cells and decreased T regulatory (Treg) cells. Dietary IPA supplementation in LSHTN mice for 3 weeks resulted in decreased systolic blood pressure, along with increased total 24 h and fractional sodium excretion. Kidney immunophenotyping demonstrated decreased Th17 cells and a trend toward increased Treg cells in IPA-supplemented LSHTN mice. In vitro, naïve T cells from control mice were skewed into Th17 or Treg cells. The presence of IPA decreased Th17 cells and increased Treg cells after 3 days. These results identify a direct role for IPA in attenuating renal Th17 cells and increasing Treg cells, leading to improved sodium handling and decreased blood pressure. IPA may be a potential metabolite-based therapeutic option for hypertension.

## 1. Introduction

Hypertension is a public health crisis that puts patients at risk for cardiovascular disease and premature death. In the United States, 1 in every 2 adults has hypertension and 1 in 3 hypertensive adults are unaware they have the condition [1,2]. As there is not a known cure for hypertension, current treatments are designed to stabilize and maintain blood pressure. The effectiveness of available treatments is somewhat limited; only approximately 24% of hypertensive American adults have their blood pressure under control [3]. Meanwhile, hypertension-related deaths have continued to rise [4,5].

In recent years, the microbiome has attracted the attention of investigators due to its undeniable impact on health and disease. The microbiome comprises bacteria, viruses, fungi, and microbes that bolster our health through various means including the production of metabolites that can modify metabolism and immune system functions. In addition to maintaining our health on a daily basis, the microbiome can also play a role in disease progression through the production of deleterious metabolites. Recent studies have reported a strong association between dysbiosis in microbiota and hypertension pathology and progression [6,7,8]. Advancements in metabolomic approaches have identified several classes of metabolites that are associated with hypertension, namely short chain fatty acids and trimethylamine-N-oxide [9,10,11,12,13,14,15,16]. Emerging evidence also shows a strong association between some tryptophan (TRP)-derived metabolites and cardiovascular disease [17,18]. TRP is a dietary essential amino acid required for many metabolic functions. Host-derived indoleamine-2,3-dioxygenase (IDO) and tryptophan-2,3-dioxygenase (TDO) transform TRP into kynurenine metabolites that have anti-inflammatory properties and can modulate cardiovascular disease pathology by activating the immune system, inducing inflammation, and exacerbating oxidative stress [19,20,21,22,23].

In addition, TRP is a precursor to several microbiota-derived metabolites, including serotonin (5-hydroxytryptamine), tryptamine, and melatonin. Bacterial metabolism or catabolism of TRP yields a variety of derivatives, such as indole, indoxyl sulphate, and indole propionic acid (IPA), to name a few [24,25]. A number of microbiota-derived TRP metabolites are decreased during microbiota dysbiosis caused by inflammation, diet, chemotherapy, antibiotic use, and aging [26,27,28,29,30]. IPA has been reported to suppress fibrosis and inflammation both at the cellular and tissue levels [31,32]. Additionally, IPA plays a neuroprotective role in Alzheimer’s disease and modulates the severity of different metabolic disorders and cardiovascular diseases [16,17,33,34,35,36,37]. As a known antioxidant, it is possible that IPA could improve outcomes in other disease pathologies.

In the current study, we hypothesized that IPA levels would be decreased significantly in serum and fecal samples from mice with L-arginine methyl ester hydrochloride (L-NAME)/high salt diet-induced hypertension (LSHTN), along with a concomitant increase in renal pro-inflammatory T helper 17 (Th17) cells and a decrease in renal anti-inflammatory T regulatory (Treg) cells. Additionally, we hypothesized that oral therapeutic supplementation with IPA during the 3-week high salt diet would decrease renal Th17 cells, increase renal Treg cells, improve renal sodium handling, and decrease blood pressure in LSHTN mice.

## 2. Results 

### 2.1. LSHTN Induces Significant Changes in Tryptophan Metabolites

**Figure 1:**
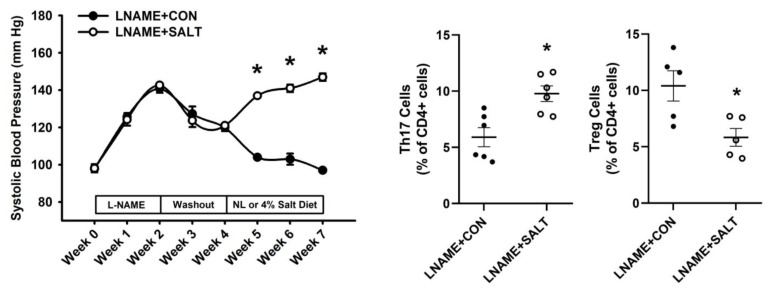
L-NAME/high salt diet-induced hypertension (LSHTN) is associated with increased T helper 17 (Th17) cells and decreased regulatory T (Treg) cells: Systolic blood pressure (SBP) measurements and Th17 and Treg cell populations from LSHTN mice and littermate controls. Results are expressed in either a line graph with mean ± SEM or in dot plots with mean (*n* = 4–6 per group). Statistical analyses were performed with a Student’s *t*-test for SBP measurements and with a Mann–Whitney U test for flow cytometric data. * *p* < 0.05 vs. LNAME + CON mice.

**Figure 2:**
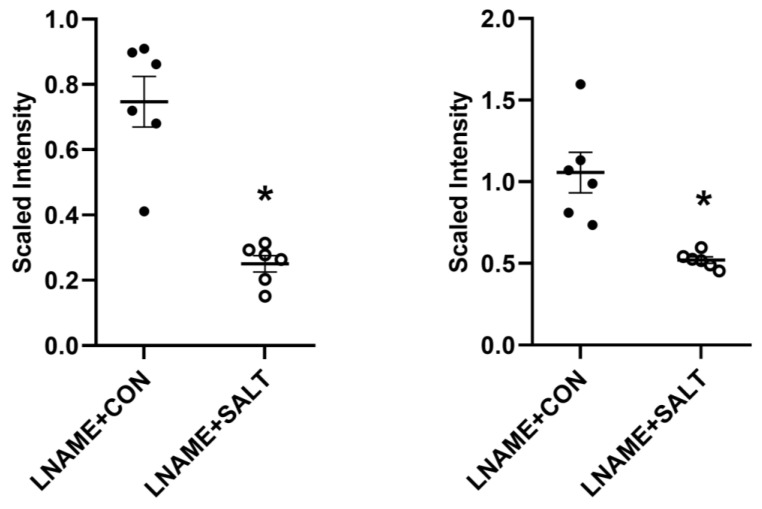
LSHTN is associated with decreased serum and fecal indole propionic acid (IPA) levels: Serum (**left**) and fecal (**right**) IPA fold changes from LSHTN mice (open circles) and littermate controls (closed circles). Results are expressed in dot plots with mean (*n* = 6 per group) and statistical analyses were performed with a Student’s *t*-test. * *p* < 0.05 vs. LNAME + CON.

Confirming our previous results in the LSHTN model, we observed that systolic blood pressure (SBP) was increased significantly for each of the 3 weeks of high salt diet (Figure 1) [38]. Flow cytometric analysis revealed that renal Th17 cells were increased significantly in LSHTN mice (LNAME + SALT) compared to control mice (LNAME + CON; Figure 1). Additionally, renal Treg cells were decreased significantly in LSHTN mice (Figure 1). Next, serum and feces samples from LSHTN mice and control mice were processed for targeted metabolic analysis where we identified that, when compared to control mice, LSHTN mice had significantly decreased IPA levels in both serum (mean ± SEM: 0.25 ± 0.03 vs. 0.75 ± 0.08) and feces (mean ± SEM: 0.52 ± 0.02 vs. 1.1 ± 0.12; Figure 2).

### 2.2. Dietary IPA Supplementation Decreases SBP in LSHTN Mice

**Figure 3:**
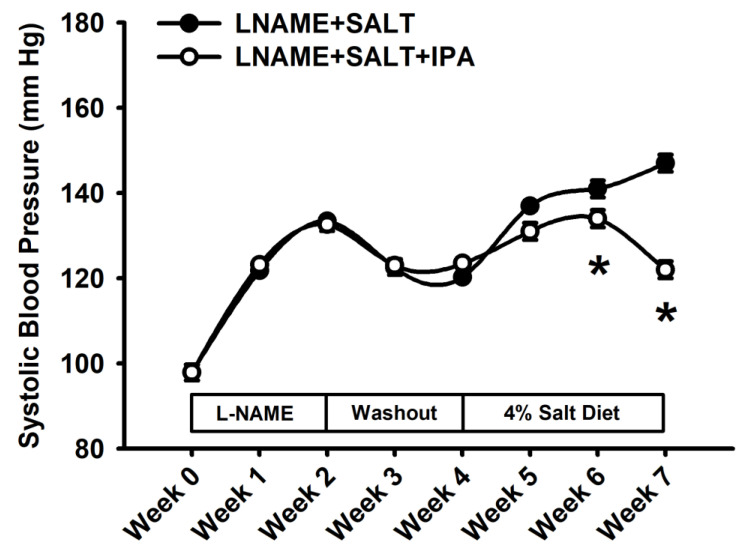
Supplementation of IPA decreases SBP in mice with LSHTN: Systolic blood pressure measurements from LSHTN mice and IPA-treated LSHTN mice. Results are expressed in a line graph with mean ± SEM (*n* = 4 per group) and statistical analyses were performed with a Student’s *t*-test. * *p* < 0.05 vs. LNAME + SALT mice.

Given the decreased serum and fecal IPA levels in LSHTN mice and the reported regulatory properties of IPA, we hypothesized that supplementing LSHTN mice with IPA would decrease blood pressure and attenuate the renal effects of hypertension in LSHTN mice. LSHTN groups were created and received either 4% salt diet (LNAME + SALT) or an IPA-supplemented 4% salt diet (LNAME + SALT + IPA). To investigate whether IPA itself has an effect on SBP, sodium handling, and immune cell populations, one control group received standard chow (LNAME + CON) and the other received IPA-supplemented chow (LNAME + CON + IPA). We observed no differences in body weight at week 3 of the diet between groups (Supplemental Figure S1). Food and water intake were not significantly different between the groups (Supplemental Figure S1). Similarly, no differences were measured in the kidney or spleen mass between the groups (Supplemental Figure S2). Two weeks after adding IPA into the food of LNAME + SALT + IPA mice, we observed a significant decrease in SBP when compared to that of LNAME + SALT mice (mean ± SEM: 134 ± 1.0 vs. 141 ± 1.4 mmHg; Figure 3). This finding persisted through week 3, as the LNAME + SALT + IPA SBP continued to decrease in comparison to the SBP of LNAME + SALT mice (mean ± SEM: 122 ± 1.5 vs. 147 ± 2.3 mmHg; Figure 3). In the absence of the salt diet to drive hypertension, IPA had no significant effect on SBP (Supplemental Figure S3).

### 2.3. Dietary IPA Supplementation Improves Renal Sodium Handling in LSHTN Mice

**Figure 4:**
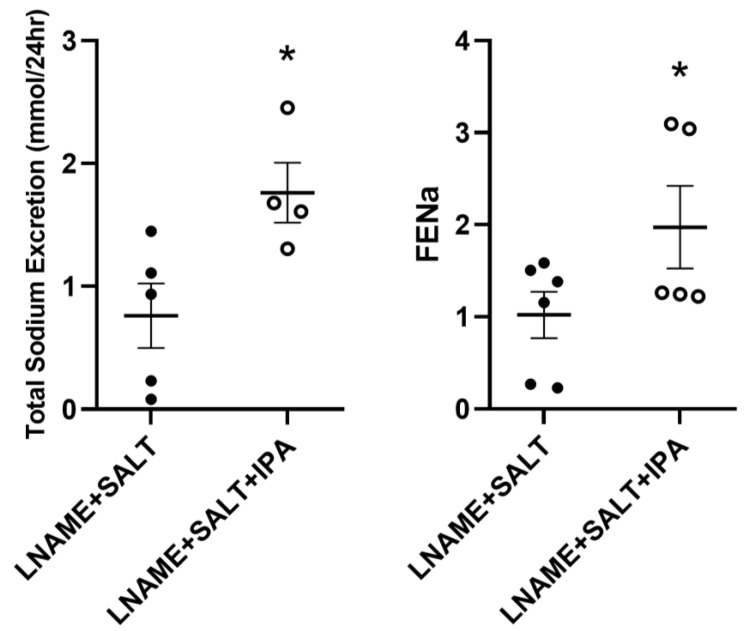
Supplementation of IPA significantly increases urinary total sodium excretion and fractional excretion of sodium (FENa) in mice with LSHTN: Urinary total sodium excretion and FENa measurements from LSHTN mice (closed circles) and IPA-treated LSHTN mice (open circles). Results are expressed as dot plots with mean (*n* = 4–6 per group) and statistical analyses were performed with a Student’s *t*-test. * *p* < 0.05 vs. LNAME + SALT mice.

During the 24 h urine collection period, LNAME + SALT + IPA mice excreted significantly more sodium than LNAME + SALT mice (mean ± SEM: 1.8 ± 0.2 vs. 0.8 ± 0.3 mmol/day; Figure 4). There were no significant differences in urinary sodium, creatinine, potassium, or chloride concentrations between groups (Supplemental Table S1). Additionally, there were no differences in urinary sodium–potassium ratios between groups (Supplemental Table S1). IPA supplementation had no effect on total sodium excretion but increased fractional excretion of sodium (FENa) in normotensive LNAME + CON mice (Supplemental Figure S4). LNAME + SALT + IPA mice had a significant increase in FENa when compared to LNAME + SALT mice (mean ± SEM: 2.0 ± 0.4 vs. 1.0 ± 0.3; *p* = 0.08; Figure 4).

### 2.4. Dietary IPA Supplementation Modulates Renal Th17 and Treg Immune Cell Populations

**Figure 5:**
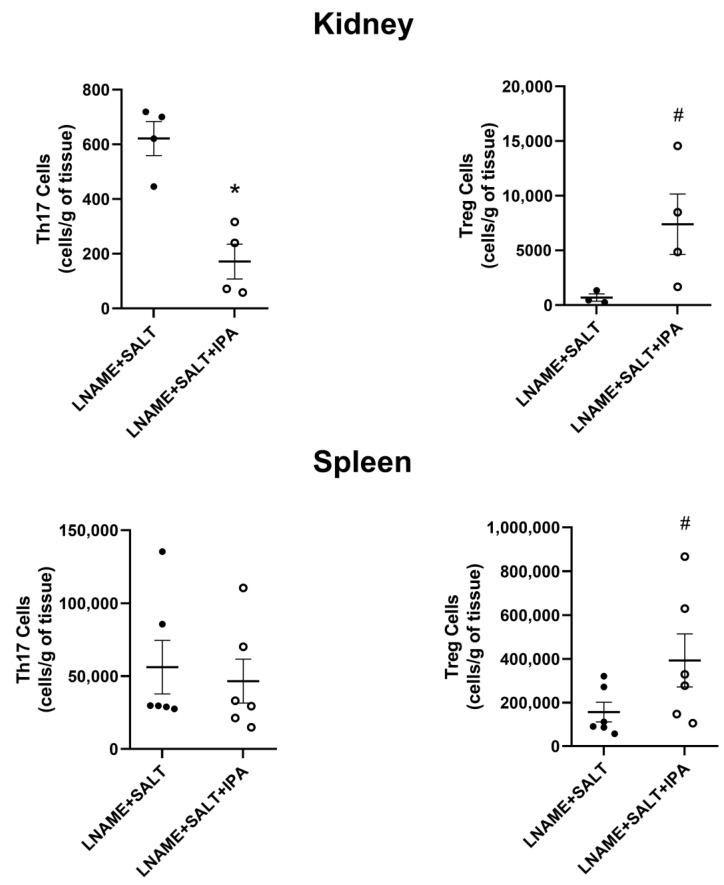
Supplementation of IPA decreases renal Th17 cells and increases renal and splenic Treg cells in mice with LSHTN: Renal and splenic Th17 and Treg cell populations in LSHTN mice (closed circles) and IPA-treated LSHTN mice (open circles). Results are expressed as dot plots with means (*n* = 3–6 per group) and statistical analyses were performed with a Mann–Whitney U test. * *p* < 0.05 vs. LNAME + SALT mice. # *p* < 0.1 vs. LNAME + SALT mice.

As we and others have previously reported, hypertensive mice typically have elevated pro-inflammatory T cells and decreased anti-inflammatory T cells that contribute to the elevation of blood pressure. Therefore, we examined T cells from hypertensive mice treated with IPA or not. At euthanasia, kidneys and spleens were processed for single cells and stained for flow cytometry (antibody panels are described in Supplemental Table S2 and in vivo gating strategies can be viewed in Supplemental Figure S5). LNAME + SALT + IPA mice had significantly decreased renal Th17 cells when compared to LNAME + SALT mice (Figure 5). Additionally, LNAME + SALT + IPA mice had elevated renal Treg cells that trended towards significance compared to the LNAME + SALT group (*p* = 0.06; Figure 5). There were no differences in splenic Th17 cells, though Treg cells trended towards an increase (*p* = 0.06; Figure 5). There were no differences in renal or splenic Th17 and Treg cell populations in control groups (Supplemental Figure S6). No significant changes between groups were observed in other renal T cell subpopulations, including naïve T cells, effector T cells, and memory T cells (Supplemental Figure S7). Similarly, there were no significant changes observed in splenic helper T cells, naïve T cells, effector T cells, or memory T cells (Supplemental Figure S8). These results suggest that in one mechanism, IPA decreases the progression of LSHTN via the improvement of renal Th17 and Treg cells. Therefore, we tested the direct effects of IPA on the development of pro- and anti-inflammatory CD4+ T cells in vitro.

### 2.5. IPA Directly Decreases Th17 Cell and Increases Treg Cell Polarization In Vitro

**Figure 6:**
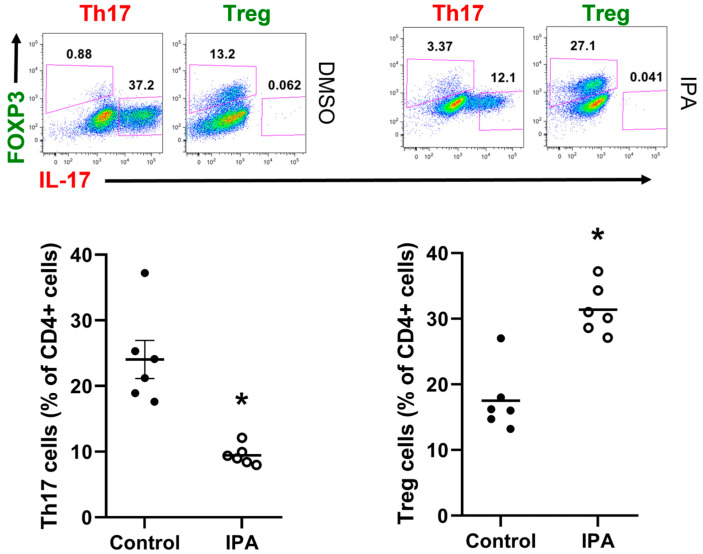
IPA presence during T cell polarization decreases Th17 cells and increases Tregs: Th17 and Treg cell populations following differentiation with (open circles) and without IPA present (closed circles). Treg cells are gated as FoxP3+ IL-17- cells and Th17 cells are gated as FoxP3- IL-17+ cells, as depicted by pink boxes. Results are expressed as dot plots with means (*n* = 6 per group) and statistical analyses were performed with a Student’s *t*-test. * *p* < 0.05 vs. control.

To test whether IPA had a direct effect on T cell phenotype, naïve CD4+ CD25- T cells were isolated from spleens of normotensive C57BL6/J mice and plated for culture with media containing cytokines for polarization into Th17 or Treg cells. Both cell types were differentiated in the presence or absence of IPA for 3 days. Cells were stained with fluorescent-conjugated antibodies against CD4, IL-17, and FoxP3 and analyzed via flow cytometry. IPA treatment caused a significant decrease in polarized Th17 cells and a significant increase in polarized Treg cells (Figure 6). These results suggest that IPA can directly antagonize the development of pro-hypertensive Th17 cells while promoting the development of anti-hypertensive Treg cells, which would be one critical pathway for improving renal sodium handling and controlling blood pressure in salt-sensitive hypertension.

## 3. Discussion

In the current study of mice with salt-sensitive hypertension, through the combination of IPA-targeted metabolomic profiling, IPA supplementation of hypertensive mice in vivo, and IPA treatment of CD4+ T cells during differentiation in vitro, we were able to uncover the beneficial role of IPA in blood pressure regulation. One of the major findings of the current study is the significant reduction in IPA levels in both serum and fecal samples from mice with LSHTN when compared to control mice. Given this, we investigated whether supplementing IPA in the diets of hypertensive mice could modulate blood pressure and impact pro-inflammatory Th17 cells, anti-inflammatory Treg cells, and renal sodium handling. Our results demonstrate that IPA supplementation decreased SBP and is accompanied by a decrease in renal Th17 cells, while Treg cells trended towards an increase in kidneys and spleens, and renal sodium handling improved in mice with LSHTN. Additionally, we demonstrate that IPA treatment reduced Th17 cells and increased Treg cells directly when naïve T cells were polarized in vitro. The present study suggests that dietary IPA supplementation reduces blood pressure in LSHTN mice, in part, by attenuating renal pro-inflammatory Th17 cells, increasing renal anti-inflammatory Treg cells, and increasing urinary sodium excretion.

Previous studies have demonstrated that supplementation of TRP reduces blood pressure in hypertensive humans and animals [39,40,41,42,43,44]. Recently, IPA has garnered interest among investigators due to its anti-inflammatory and antioxidant potential [25,32,45]. One of the prominent observations in the current study is a significant decrease in IPA levels in both serum and fecal samples from hypertensive mice. Supplementation of IPA in the diet lowered blood pressure in hypertensive mice. In contrast to these results, IPA has been reported to increase blood pressure in normotensive rats and was associated with increased contractility of heart muscles [46,47]. In an ex vivo setup, IPA was reported to promote vasoconstriction of endothelium-denuded mesenteric resistance arteries (MRA) isolated from normotensive rats [46]. Notably, vasodilation of MRA is dependent on nitric oxide derived from the endothelium, and IPA has been reported to prevent nitric-oxide-dependent dilation of aorta and pulmonary arteries [48,49]. Interestingly, the beneficial role of IPA was confirmed through the increased metabolic activity of cardiomyocytes in vitro [46]. These inconsistencies could be due to the use of different animal models along with differences in the route of administration, duration, and dosage of IPA. Additionally, decreased IPA levels have been associated with colitis, diabetes, obesity, and atherosclerosis [18,50,51,52,53]. Altogether, our results and studies carried out by other authors support our hypothesis that IPA administration attenuates blood pressure in hypertensive mice. To the best of our knowledge, this is the first study to establish an inverse correlation between IPA levels and blood pressure in hypertensive mice.

Hypertension is well-known to be associated with an infiltration of pro-inflammatory immune cells, including Th17 cells, into the kidney [38,54]. Studies have reported an imbalance of anti-inflammatory Treg cells and pro-inflammatory Th17 cells in hypertension [55]. TRP fills an immunomodulatory role by promoting differentiation of Treg cells and reducing Th17 cells [56]. TRP metabolites, including IPA, indole-3-aldehyde, indole-3-acid-acetic, and indole-3-acetaldehyde, serve as ligands for the aryl hydrocarbon receptor (AhR) [51,57]. A number of distinct AhR ligands can activate or suppress Treg cells and Th17 cells both in vitro and in vivo, which suggests further high-resolution structure-activity studies are needed [58,59,60]. Administration of IPA attenuates pro-inflammatory (CD4+IFNγ+IL10−) T cells and increases anti-inflammatory (CD4+IFNγ−IL10+) T cells in the colon in a mouse model of colitis [61]. An in vitro study also reported that IPA directly induces differentiation of Treg cells [61]. In line with these studies, supplementation of IPA in the present study reduced renal Th17 cells while tending to increase Treg cells in mice with LSHTN, and decreased Th17 cells and increased Treg cells directly in vitro, providing strong evidence that this metabolite may share the biological effects of its amino acid precursor.

Reduced IPA levels have been associated with decreased renal function and chronic kidney disease [62]. Administration of IPA inhibited the expression of fibrotic and inflammatory markers in renal proximal tubular cells [31]. Similarly, in this study, we demonstrated that IPA supplementation decreases renal pro-inflammatory Th17 cells, increases renal anti-inflammatory Treg cells, and increases urinary total sodium excretion and FENa in mice with LSHTN. It is possible that the anti-inflammatory effects of IPA improved renal sodium handling, which contributed to the reduced blood pressure in the hypertensive mice. It is also possible that IPA affected blood pressure in our study by reducing oxidative stress, as drugs with antioxidant properties are able to decrease blood pressure.

Nonetheless, these findings confirm IPA as a promising therapeutic for the management of blood pressure in hypertensive patients with salt sensitivity. Further investigations are warranted on the molecular mechanisms through which metabolites interact with the host immune system, and on whether these contribute to the pathogenesis of hypertension or attenuate it.

## 4. Materials and Methods

### 4.1. Statistics

Results are presented as line graphs with mean ± SEM or dot plots with means. Metabolic profile data were analyzed with Welch’s *t*-test to determine differences between LNAME + SALT and LNAME + CON mice. For all other aspects of the study, the differences between groups were determined by 2-tailed unpaired Student’s *t*-tests, with the significance level set at 0.05 for all comparisons. When the threshold for normality was not met, nonparametric Mann–Whitney U tests were used to determine statistical significance, with the significance level set at 0.05. All statistical analyses were performed using SigmaPlot 10.0 software (Systat, San Jose, CA, USA) and GraphPad Prism 8.0.1 software (Dotmatics, Boston, MA, USA).

### 4.2. Mice

C57BL/6J male mice were purchased from Jackson Laboratories (Bar Harbor, ME, USA). All mice received water and food ad libitum at all stages, regardless of group assignment. The mice were euthanized following the 3-week diet phase via axillary vein exsanguination under 5% inhaled isoflurane anesthesia, with death confirmed through cervical dislocation prior to tissue collection.

#### 4.2.1. L-NAME/High Salt Diet-Induced Hypertension (LSHTN)

Mice assigned to LSHTN groups were made hypertensive by providing these with L-NAME (0.5 mg/mL; Sigma, St. Louis, MO, USA) in their drinking water for 2 weeks. After a 2-week washout period where all mice received regular drinking water, these mice were fed a 4% high salt diet for 3 weeks as previously described by us and others.

#### 4.2.2. Metabolomic Profile Analysis

At 10 weeks of age, mice were made hypertensive as described above (LNAME + SALT; *n* = 6). Littermate control mice underwent the L-NAME priming period and washout period but received regular chow with a salt content of approximately 0.5% (Teklad 8604; Envigo, Indianapolis, IN, USA) during the diet phase (LNAME + CON, *n* = 6).

#### 4.2.3. IPA Supplementation

At 10 weeks of age, the mice were assigned to one of four treatment groups: two LSHTN groups and two control groups (*n* = 6 per group). Mice assigned to the LNAME + SALT group were given LSHTN as described above, whereas mice assigned to the LNAME + SALT + IPA group received 562.5 mg/kg body weight IPA (Sigma) added to their 4% salt chow during the diet phase. Control groups were generated to control for diet (LNAME + CON) and IPA treatment (LNAME + CON + IPA). LNAME + CON mice underwent L-NAME priming and a washout period but received regular chow during the diet phase. LNAME + CON + IPA mice also underwent L-NAME priming and a washout period as described above but received regular chow with 562.5 mg/kg IPA during the diet phase. Over the course of treatments, food and water intake were monitored for each cage. These data were averaged to each mouse and can be viewed in Supplemental Figure S1.

### 4.3. Blood and Fecal Collection

Immediately prior to euthanasia, the mice were housed individually in autoclaved cages to allow for the collection of fecal samples. The samples were removed immediately and flash-frozen in liquid nitrogen prior to storage at −80 °C. Blood was collected via the left ventricle and kept on ice. After 30 min of clotting, samples were centrifuged at 6000 RPM for 6 min. Serum was collected and stored at −80 °C until analysis. The samples were shipped overnight in dry ice to Metabolon, Inc. (Morrisville, NC, USA) for IPA-targeted metabolomic analysis.

### 4.4. Blood Pressure

SBP was determined weekly using the tail-cuff method after appropriate acclimatization and training as we have published previously. All measurements were acquired using the IITC Life Science noninvasive blood pressure acquisition system (IITC Inc., Woodland Hills, CA, USA). After 30 min of acclimatization in a designated dark, quiet area, mice were loaded into pre-warmed restrainers and tail cuffs and allowed an additional 5–10 min to acclimate inside the 34 °C warmed chamber. SBP was derived from pressure tracings carried out by two independent, blinded investigators.

### 4.5. Urine Analysis

Prior to euthanasia, the mice were acclimated to single capacity metabolic cages (Hatteras, Cary, NC, USA) overnight. The following day, urine collection tubes were replaced, and 24 h collection was initiated. This collection period concluded immediately prior to euthanasia. Samples were briefly centrifuged to remove debris and stored at −80 °C until analysis. Urine samples were analyzed via capillary electrophoresis for sodium, potassium, and chloride using a DxC 700 AU Chemistry Analyzer (Beckman Coulter, Brea, CA, USA). Urinary creatinine concentrations were calculated through direct potentiometry using a P/ACE MDQ Plus Capillary Electrophoresis System (Sciex, Redwood City, CA, USA). Urinary sodium–potassium ratios, total sodium excretion, and fractional excretion of sodium (FENa) were calculated using the values outlined above.

### 4.6. Flow Cytometry

Kidneys and spleens were collected at euthanasia and immediately transferred into RPMI media on ice. Kidneys were homogenized for 35 min at 37 °C in RPMI media with enzymes from a Multi Tissue Dissociation Kit 2 (Miltenyi Biotec, Bergisch Gladbach, Germany). Spleens were homogenized for 1 min at room temperature in RPMI media. All tissues were homogenized with constant disruption provided by a gentleMACS^TM^ Octo Dissociator (Miltenyi). Tissue homogenates were filtered and rinsed through 100- and 40-micron strainers. Then, red blood cells were lysed in ACK Lysing Buffer (Life Technologies, Carlsbad, CA, USA). At this point, samples were resuspended in 1 mL PBS and 100 µL aliquots of kidney cells and 50 µL aliquots of spleen cells were incubated with Ghost Dye Violet 510 (Tonbo Biosciences, San Diego, CA, USA) for 30 min at 4 °C. To prevent nonspecific binding, the cells were resuspended in 0.1% FBS solution and incubated with anti-mouse CD16/CD32 (BD Biosciences, San Jose, CA, USA) for 10 min at 4 °C. Then, the cells were incubated with fluorescently conjugated antibodies against CD45, CD4, CD25, CD44, and CD62L (see Supplemental Table S2 for panel descriptions). Afterwards, cells underwent intracellular staining using a Foxp3/Transcription Factor Staining Buffer Set (eBioscience, Inc., San Diego, CA, USA). These cells were incubated with antibodies against FoxP3, TNFa, and IL17 for 30 min at 4 °C. After all staining was completed, cells were washed, resuspended in 0.1% FBS solution, and filtered through 35-micron strainers. Data were acquired on an LSRFortessa X-20 flow cytometer equipped with FACSDiva v9.0 software (BD Biosciences). Cell populations were analyzed using FlowJo v10.1 (FlowJo, LLC, Ashland, OR, USA). Antibody compensation controls were prepared from UltraComp eBeads Compensation Beads and viability dye compensation controls were prepared from an ArC Amine Reactive Compensation Bead Kit (Invitrogen, Carlsbad, CA, USA).

### 4.7. Cells

#### 4.7.1. Isolation of Splenic CD4+ Naive T Cells

C57BL6/J mice were euthanized, and spleens were harvested, homogenized in RPMI, and filtered and rinsed through 70-micron strainers. Red blood cells were lysed using ACK Lysing Buffer (Life Technologies) and non-specific binding prevented via incubation with anti-mouse CD16/CD32 (BD Biosciences). Cells were first stained with Ghost Dye Violet 510 viability dye (Tonbo Biosciences) and then stained with fluorescently conjugated antibodies against CD4 and CD25 (antibody information listed in Supplemental Table S2). Live CD4+ CD25- naïve CD4+ T cell populations were isolated from the cell suspension using a FACSAria II Cell Sorter (BD Biosciences) equipped with a 70-micron nozzle and FACSDiva v9.0 acquisition software.

#### 4.7.2. In Vitro Modulation of T Cell Differentiation with IPA

Isolated naïve CD4+ T cell populations were added to 96-well plates (1 × 10^5^ cells per well) precoated with antibodies against CD3 (5 µg/mL) and CD28 (2 µg/mL; Bio X Cell, Lebanon, NH, USA). The cells were cultured in the presence of the cytokines TGF-β1 (0.5 ng/mL; Peprotech, Cranbury, NJ, USA) and IL-6 (10 ng/mL; Peprotech) for Th17 differentiation, or TGF-β1 (2 ng/mL) alone for Treg differentiation. The cells were differentiated in the presence of either IPA (1 mM; Sigma) or DMSO at 37 °C with 5% CO2 for 3 days. The cells were then stained with fluorescently conjugated antibodies against CD4, IL-17, and FoxP3 (antibody information listed in Supplemental Table S2). Data were acquired on an LSRFortessa X-20 flow cytometer using FACSDiva v9.0 software (BD Biosciences). FlowJo v10.1 software (FlowJo, LLC) was used for data analysis.

## Data Availability

The data, analytic methods, and study materials that support the findings of this study are available from the corresponding author upon reasonable request.

## Supplementary Materials

The following supporting information can be downloaded at: https://www.mdpi.com/article/10.3390/ijms24119192/s1.
