# Supplementary material for: Indole Propionic Acid Increases T Regulatory Cells and Decreases T Helper 17 Cells and Blood Pressure in Mice with Salt-Sensitive Hypertension
Source: Int J Mol Sci. 2023 May 24;24(11):9192. doi: 10.3390/ijms24119192 (PMC10252743; doi:10.3390/ijms24119192)
Supplement: Supplementary file 1 [file ijms-24-09192-s001.zip › ijms-2331579-supplementary.pdf]

Supplementary Materials

Indole Propionic Acid Increases T Regulatory Cells and Decreases T Helper 17 Cells and Blood Pressure in Mice with Salt Sensitive Hypertension

DISCLOSURES

Robert C. Alaniz is a founder and holds equity in Tlaloc Therapeutics Inc., which is commercializing microbiota therapeutics. The remaining authors have declared that no conflict of interest exists.

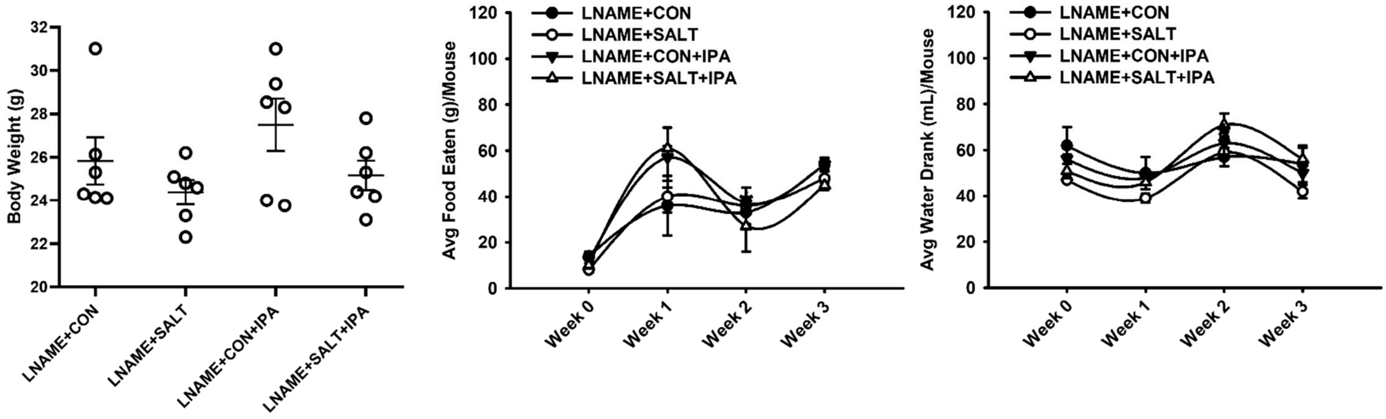

Supplemental Figure S1. Dietary IPA supplementation does not alter body weight, food intake, and water intake in LSHTN mice. Body weights, average food intake, and average water intake in littermates with and without LSHTN and with and without IPA treatment (n=6 per group). Statistical analyses between groups were performed with a Student's t-test.

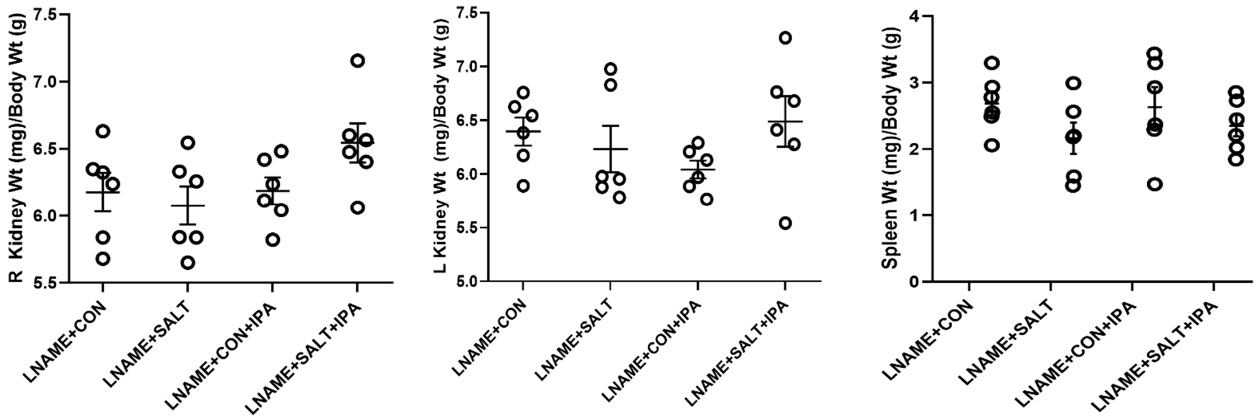

Supplemental Figure S2. Dietary IPA supplementation does not alter kidney or spleen weights in LSHTN mice. Weights of the right kidney, left kidney, and spleen in littermates with and without LSHTN and with and without IPA treatment (n=6 per group). Statistical analyses between groups were performed with Student's t-test.

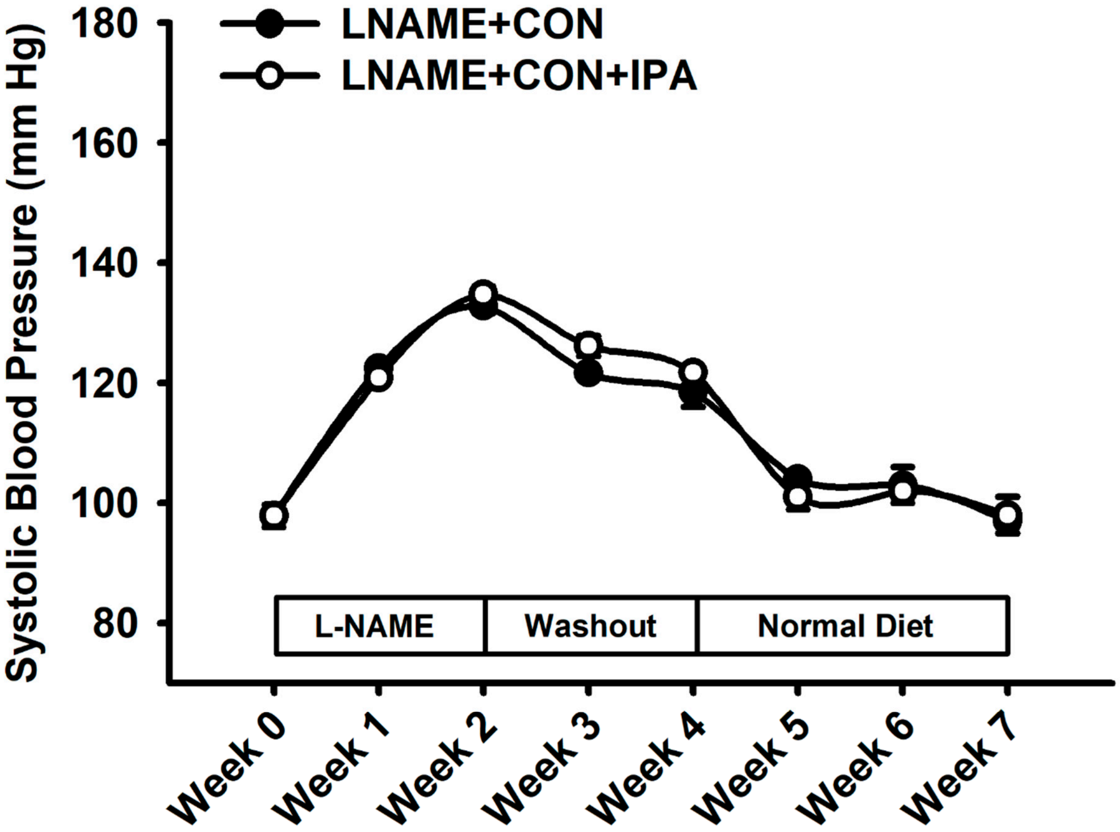

Supplemental Figure S3. In normotensive mice, dietary IPA supplementation has no effect on systolic blood pressure. Systolic blood pressure measurements in mice with and without dietary IPA treatment (n=4 per group). Statistical analyses were performed with Student's t-test.

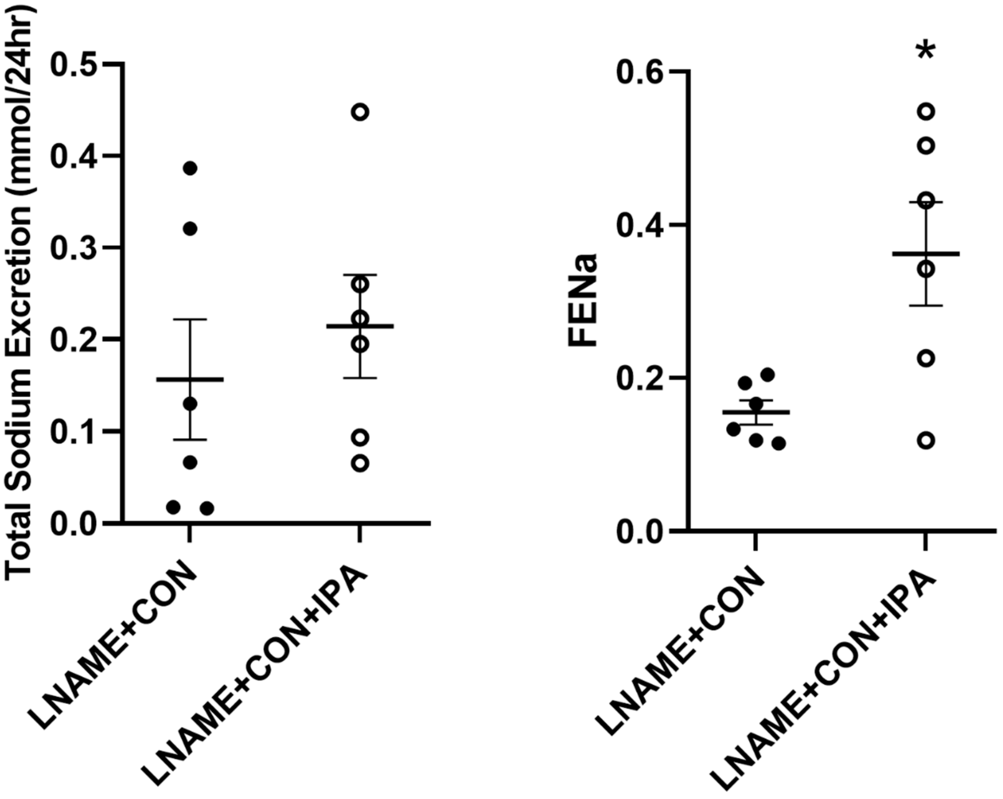

Supplemental Figure S4. In normotensive mice, dietary IPA supplementation does not alter urinary total sodium excretion and increases fractional excretion of sodium. Urinary total sodium excretion and FENa in mice with and without dietary IPA treatment (n=6 per group). Statistical analyses were performed with Student's t-test.

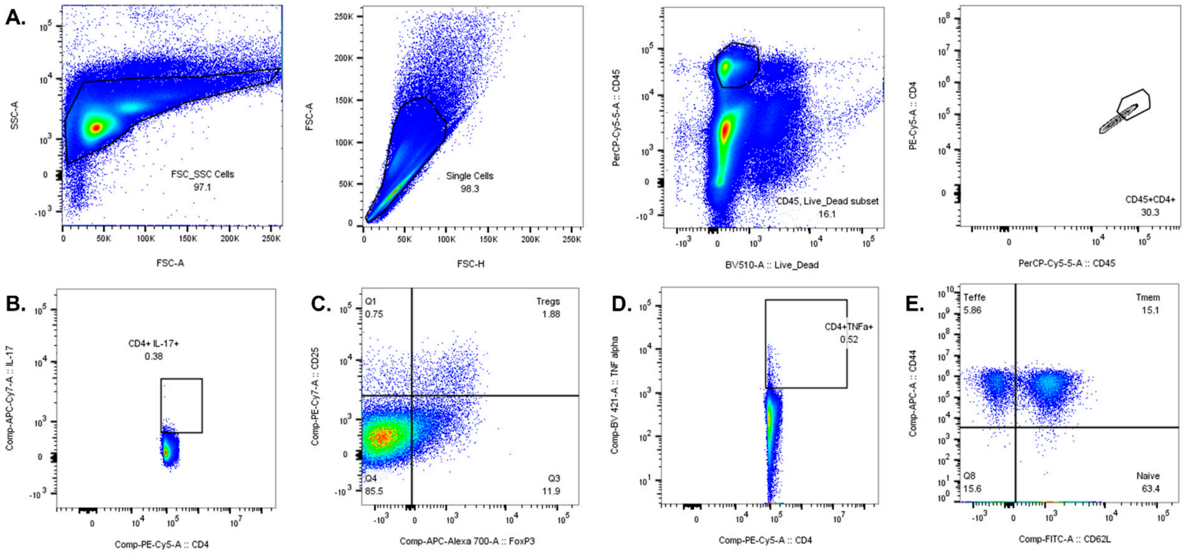

Supplemental Figure S5. Gating strategy for CD4+ T cell subsets in the kidney and spleen. (A) Cells were gated to select for live, singlet CD45+CD4+ cells. Then, cells were gated for (B) Th17 cells, (C) Treg cells, (D) T helper 1 cells, (E) naïve T cells, effector T cells, and memory T cells.

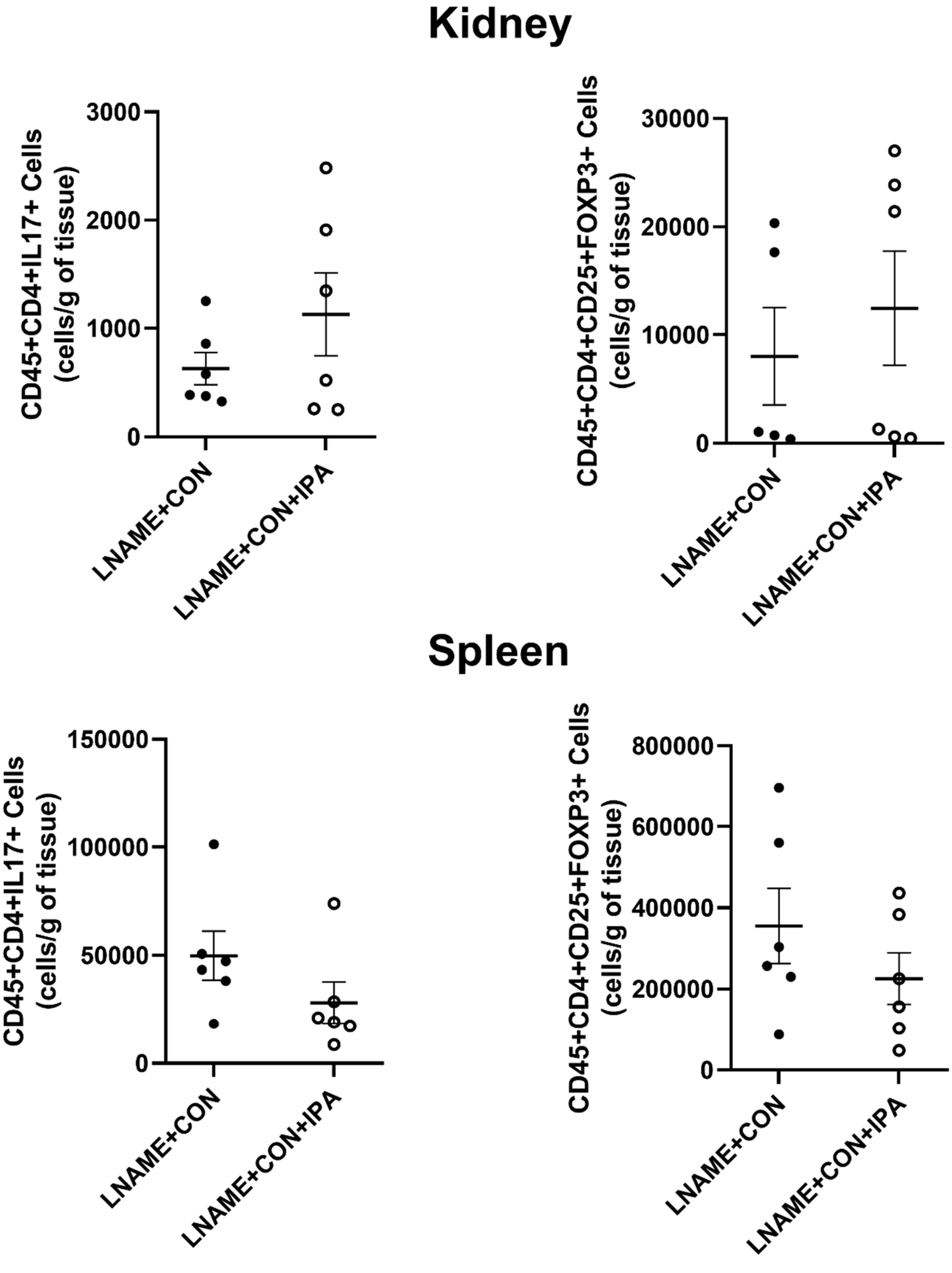

Supplemental Figure S6. In normotensive mice, dietary IPA supplementation does not alter Th17 or Treg populations in the kidney or spleen. Th17 and Treg cell populations in mice with and without dietary IPA treatment (n=6 per group). Statistical analyses were performed with Student's t-test.

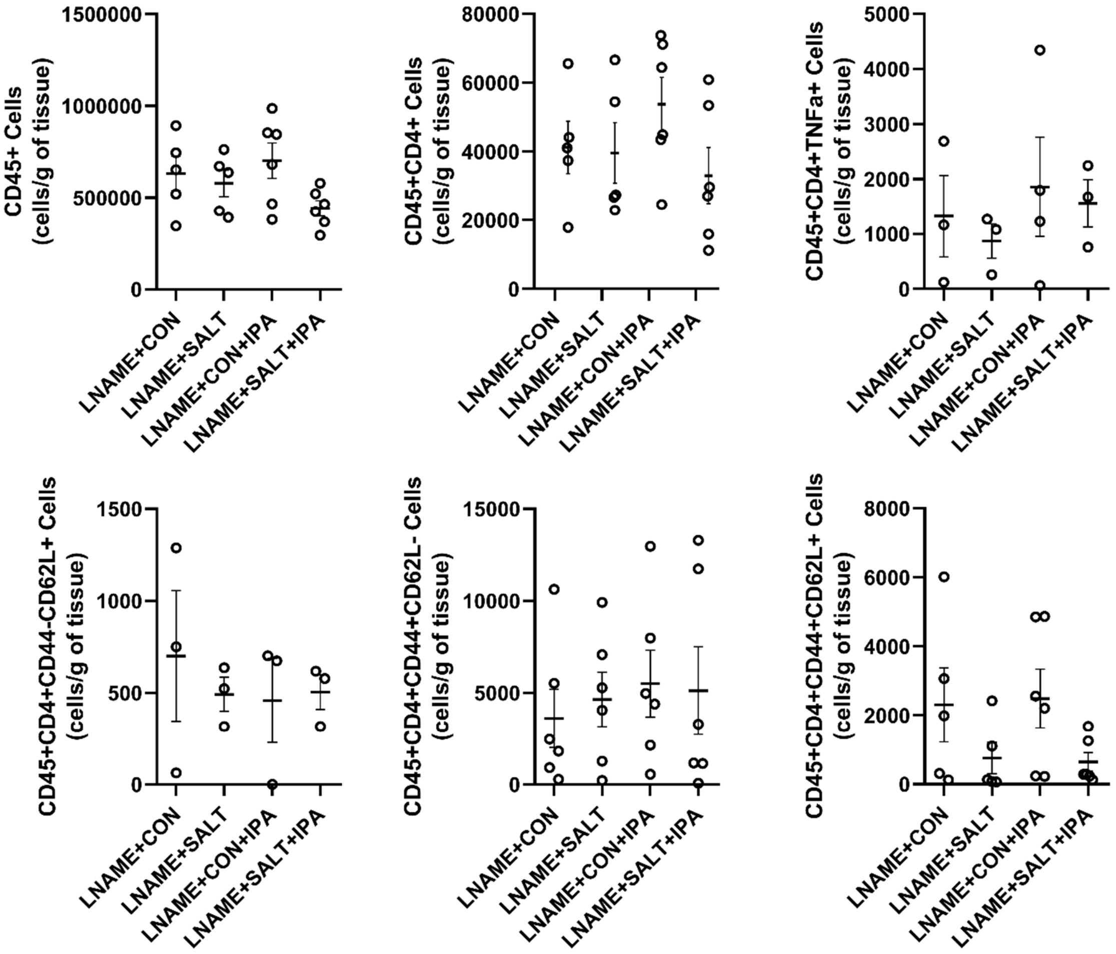

Supplemental Figure S7. Supplementation of IPA in mice with LSHTN does not alter renal T cell populations. Populations of renal immune cells, T helper cells, T helper 1 cells, naïve T cells, effector T cells, and memory T cells in mice with and without LSHTN and with and without dietary IPA supplementation (n=3-6 per group). There were no significant differences between LNAME+CON and LNAME+CON+IPA or LNAME+SALT and LNAME+SALT+IPA. Statistical analyses were performed with Student's t-test.

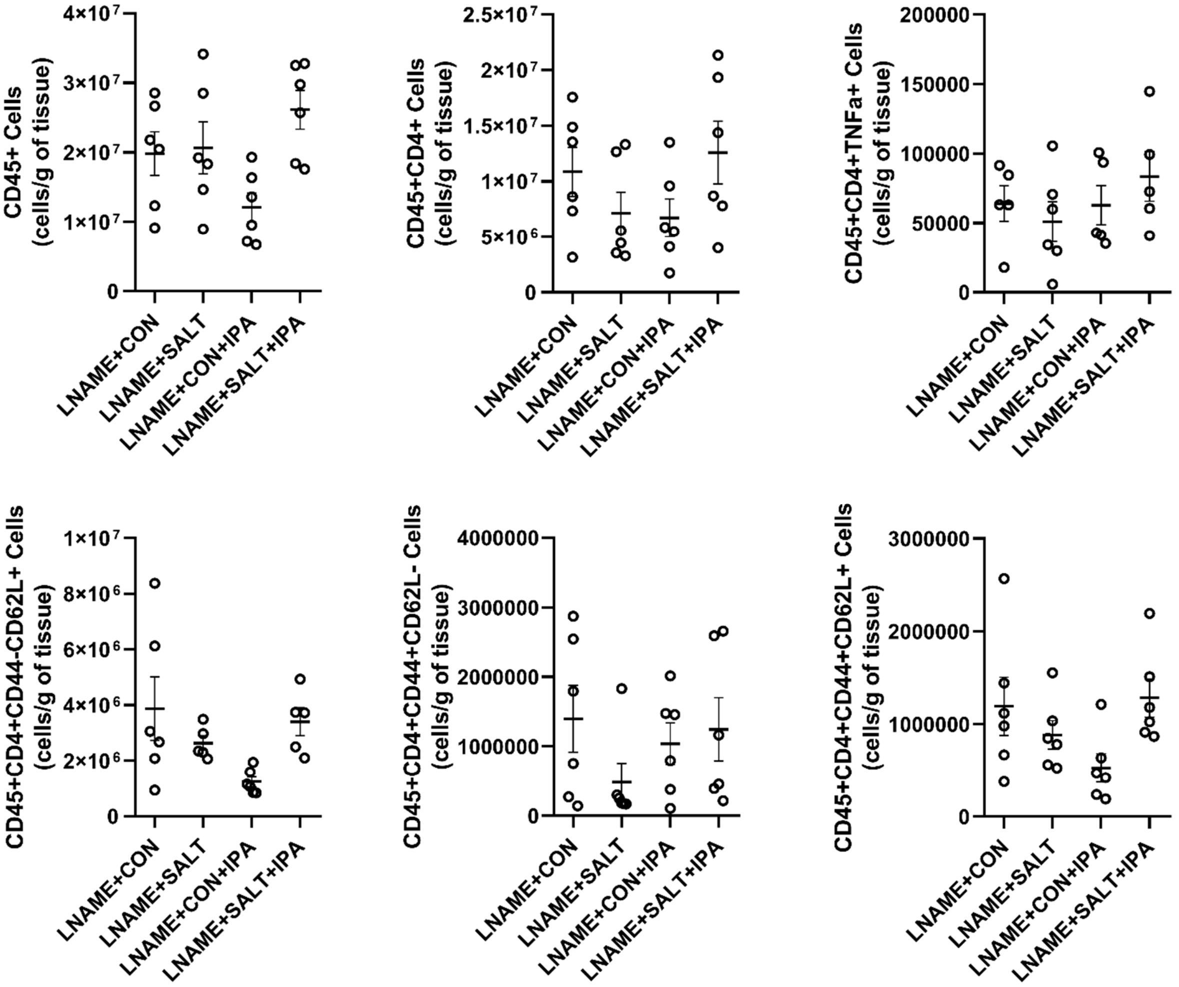

Supplemental Figure S8. Supplementation of IPA in mice with LSHTN does not alter splenic T cell populations. Populations of splenic immune cells, T helper cells, T helper 1 cells, naïve T cells, effector T cells, and memory T cells in mice with and without LSHTN and with and without dietary IPA supplementation (n=5-6 per group). There were no significant differences between LNAME+CON and LNAME+CON+IPA or LNAME+SALT and LNAME+SALT+IPA. Statistical analyses were performed with Student's t-test.

Supplemental Table S1. Urinary sodium, creatinine, potassium, and chloride concentrations and sodium:potassium ratios.

	**Sodium (mmol/L)**	**Creatinine (mg/dL)**	**Potassium (mmol/L)**	**Chloride (mmol/L)**	**Sodium:Potassium (ratio)**
**LNAME+CON**	112 ± 11	44 ± 5	278 ± 15	152 ± 13	0.4 ± 0.03
**LNAME+SALT**	503 ± 108	43 ± 8	229 ± 20	548 ± 126	2.2 ± 0.4
**LNAME+CON+IPA**	105 ± 7	32 ± 7	304 ± 20	163 ± 22	0.3 ± 0.02
**LNAME+SALT+IPA**	540 ± 50	33 ± 5	198 ± 14	504 ± 103	2.7 ± 0.2

There were no significant differences between LNAME+CON and LNAME+CON+IPA or LNAME+SALT and LNAME+SALT+IPA. Statistical analyses were performed with Student's t-test.

Supplemental Table S2. Flow cytometry antibody panel descriptions for mouse kidneys and spleens.

**Panel**	**CD4-Lineage T Cells**					
**Fluorochrome**	PerCP-Cy5.5	PE-Cy5	PE-Cy7	APC	FITC	Ghost Dye Violet 510
**Antigen**	CD45.2	CD4	CD25	CD44	CD62L	Live/dead
**Final Conc (µg/mL)**	4 (kid)<br>1 (spl)	4 (kid)<br>1 (spl)	4 (kid)<br>1 (spl)	4 (kid)<br>1 (spl)	10 (kid)<br>2.5 (spl)	
**Dilution Factor**	1:50 (kidney)<br>1:200 (spleen)					1:400
**Clone**	104	RM4-5	PC61	IM7	MEL-14	
**Manufacturer**	BD	BioLegend	BioLegend	BioLegend	BioLegend	Tonbo

**Panel**	**CD4-Lineage T Cells (Intracellular)**		
**Fluorochrome**	AlexaFluor 700	BV421	APC-Cy7
**Antigen**	FoxP3	TNFa	IL17a
**Final Conc (µg/mL)**	4 (kid)<br>1 (spl)	4 (kid)<br>1 (spl)	4 (kid)<br>1 (spl)
**Dilution Factor**	1:50 (kidney)<br>1:200 (spleen)		
**Clone**	FJK-16s	MP6-XT22	TC11-18H10.1
**Manufacturer**	eBioscience	BD	BioLegend

Abbreviations: APC = allophycocyanin; FITC = fluorescein isothiocyanate; PE = phycoerythrin; PerCP-Cy5.5 = peridinin chlorophyll protein complex cyanine 5.5
